# Chronic abdominal aortic dissection, endovascular treatment using a new Stent-graft for in situ Fenestration

**DOI:** 10.1186/s42155-021-00208-8

**Published:** 2021-01-29

**Authors:** Hernan G. Bertoni, German A. Girela, Hector D. Barone, Federico De Caso, Alejandro De La Vega, Bao T. Bui, Thomas Maldonado

**Affiliations:** 1 Department of Interventional Radiology, Fleni Institute, Buenos Aires University, Caba, Argentina; 2 Department of Cardiovascular Surgery, Laben, Rio Negro, Argentina; 3 Latecba, SA Buenos Aires, Argentina; 4 Department of Interventional Radiology, Sherbrooke University, Sherbrooke, Canada; 5 Department of Vascular Surgery, NYU University, New York, USA

**Keywords:** Aorta, Chronic Aortic Dissection, In Situ Fenestration, Stent Graft

## Abstract

**Background:**

Although endovascular treatment of the thoracic aorta (TEVAR) has become an elective procedure for treatment of complicated type B aortic dissection, its role in treating post dissection thoraco-abdominal aortic aneurysm (TAAA), is still limited. This is a case of aortic vascular disease, which reports the use of a new endovascular device.

**Case presentation:**

: We present the case of a 62 year old male patient with a history of hypertension, active smoker, who presented penetrating descending thoracic aortic ulcer in the setting of a chronic abdominal aortic dissection. The patient was treated using a new stent graft capable of in situ fenestration that allowed crossing the stent-graft membrane, implanting a covered stent to exclude the re-entry at the level of the left renal artery and redirecting the blood flow through the true lumen.

**Conclusions:**

This case report demonstrates the feasibility of a novel stent-graft concept. Larger studies with longer follow-up are essential to fully evaluate the safety and effectiveness of this new design.

## Background

Although endovascular repair of the thoracic aorta has become a promising treatment for complicated type B acute dissection, its role in treating chronic dissection with aneurysmatic dilatation of the thoraco-abdominal aorta remains limited, primarily due to persistent retrograde flow to the false lumen (FL) through re-entry tears in the abdominal aorta and / or iliac arteries [1]. Aortic dilatation is a crucial factor that negatively impacts the long-term survival of these patients [2]. The objective of this work is to demonstrate the feasibility of a new stent-graft for treatment of complex paravisceral aortic aneurysms in the setting of a chronic dissection. Unique to this design is the ability to perform in situ fenestration for the left renal artery thereby closing the re-entry flap at this level and excluding flow from the false lumen (FL).

## Case report

**Fig. 1:**
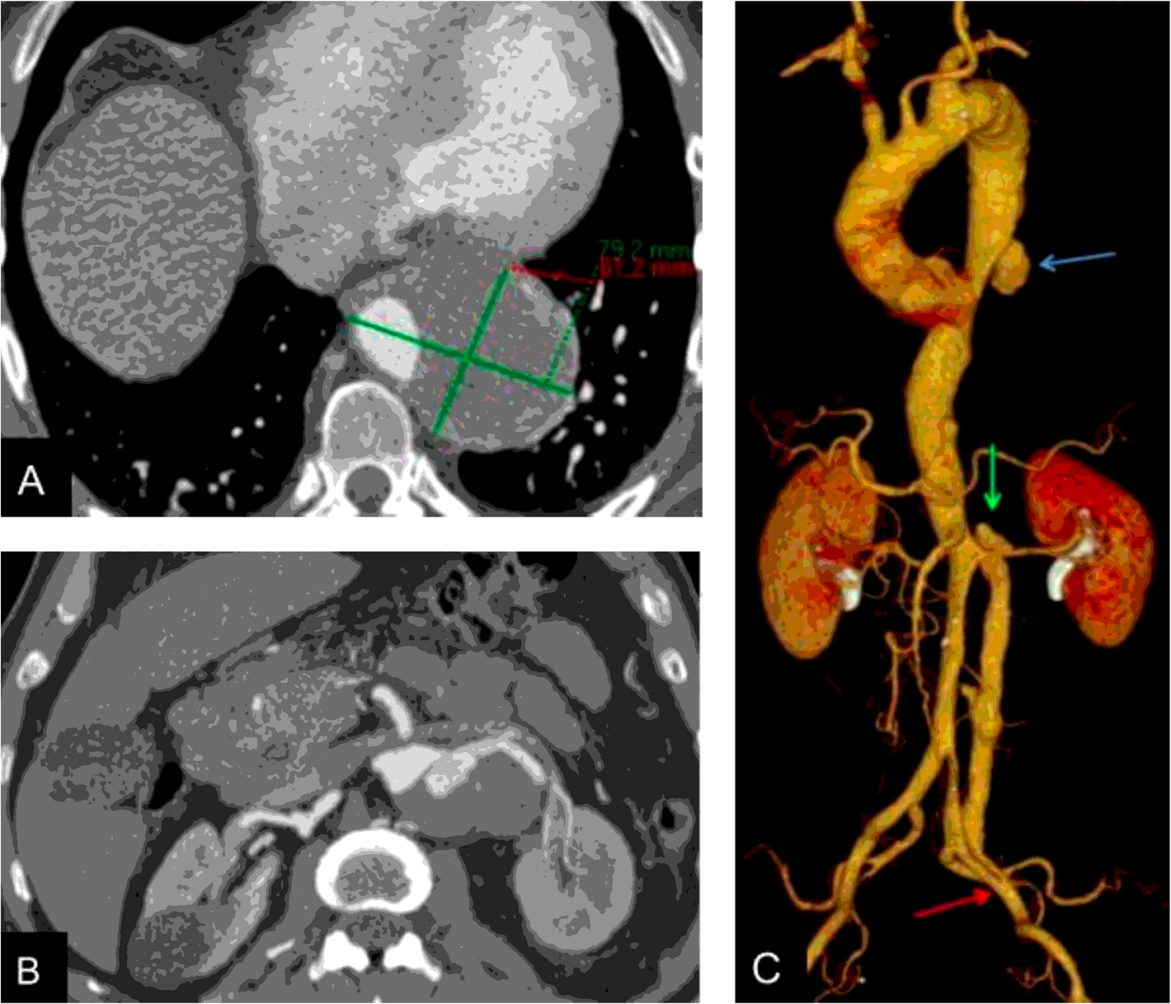
A y B. Axial contrast-enhanced image;**a** Large periaortic intramural hematoma in sigmoid aorta. **b** Left renal artery arising from the TL with proximal reentry, associated aortic aneurysm. **c** 3D reconstruction showing an aortic ulcer in the descending aorta (blue arrow), re-entry of the FL at the level of the left renal artery (green arrow), and re-entry of the FL at the level of the left external iliac artery (red arrow)

A 62-year-old male patient, active smoker with a history of hypertension, was found to have an incidental aortic aneurysm on abdominal ultrasound performed in preparation for elective cholecystectomy. Contrast enhanced CT revealed a penetrating descending thoracic aortic ulcer and associated intramural hematoma measuring 79 mm (Fig. 1a) in the setting of a as well as a chronic aortic dissection with permeable FL. The abdominal aorta measured 59 mm in greatest diameter, (Fig. 1b). The celiac trunk, superior mesenteric artery and right renal artery originated from the TL. The left renal artery originated from the TL with evidence of a re-entry tear supplying FL flow at that location. The true lumen in the infrarenal aorta was compressed and a distal re-entry was noted at the left external iliac artery (Fig. 1c).

A two-stage endovascular repair was decided upon due to the complexity of the case. First, the penetrating ulcer of the thoracic aorta was treated with two Hercules™ self-expandable stent-grafts (Micro Port. Shanghai. China) with immediate good results.

Three months later, the patient underwent the second staged endovascular repair under general anesthesia with placement of a spinal drain. Bilateral femoral arteries and the right subclavian arteries were accessed via surgical cutdown. Two 70 cm multipurpose Flexor® guiding sheaths (8Fr and 7Fr) (Cook Medical. Bloomington, IN. USA) were introduced via the subclavian artery and used to selectively catheterize the superior mesenteric artery and the right renal artery, respectively. A PTFE SICBI G SETA® (Latecba.SA. Buenos Aires. Argentina) balloon expandable covered stent (8 × 38 mm) was deployed in the superior mesenteric artery and another PTFE SICBI G SETA® (Latecba.SA. Buenos Aires. Argentina) balloon expandable covered stent (7 × 38 mm) in the right renal artery, so as to maintain blood flow to these arteries in a planned chimney procedure. The left renal artery was left unstented for the second part of the current procedure.

The right femoral access was used to deliver a 25 mm x 80 mm RAKB SETA® balloon-expandable stent-graft (Latecba.SA. Buenos Aires. Argentina) in the abdominal aorta. This is deployed carefully at low pressure until the resistance of the intima is met. Next, a 25 mm x 50 mm SETA MUG® balloon-expandable stent-graft (Latecba.SA. Buenos Aires. Argentina) was placed at the level of the renal arteries, intentionally covering the origin of the left renal artery, in preparation for in-situ fenestration procedure. Once this last aortic stent-graft was fully expanded, we expanded the previously placed stents in the superior mesenteric and right renal arteries using standard parallel grafting techniques.

**Fig. 2:**
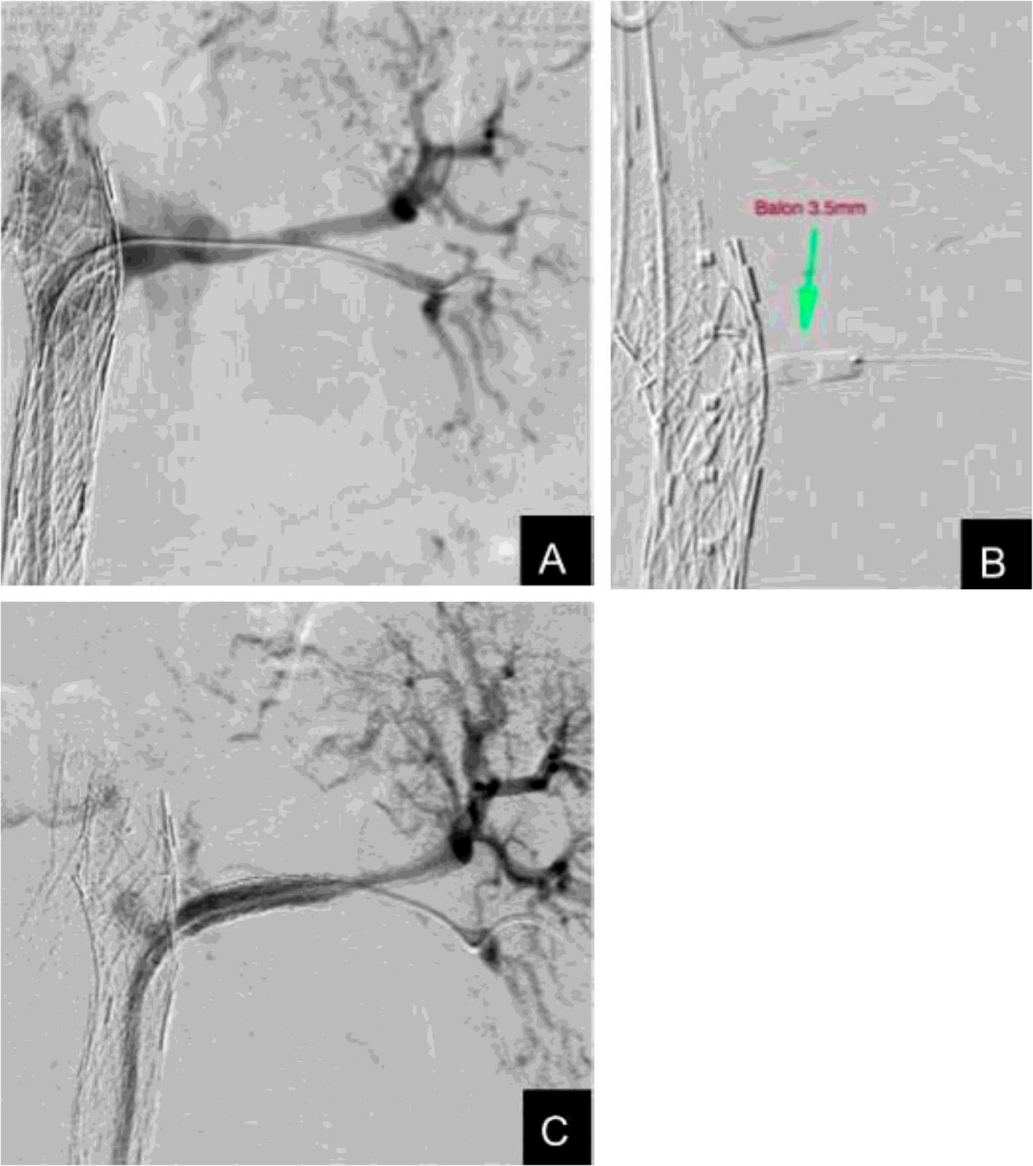
**a** Control angiography through a guide catheter that shows MUG permeability with patent FL. **b** Balloon angioplasty for the opening of the membrane. **c** Angiographic control showing reconstitution of flow through the TL with complete sealing of the FL

We then introduced an 8 Fr OSCOR® steerable guide catheter (Oscor Inc. Florida. USA) via the right femoral access, and positioned it within the SETA MUG stent graft, directly in line with the ostium of the left renal artery (Fig. 2a). Using a Terumo® 0.035” straight hydrophilic standard guide (Terumo Corp. Tokyo. Japan), the SETA MUG membrane was easily punctured and crossed, thus gaining access to the false lumen and left renal artery. This wire was exchanged for a 0.014” support guide, over which a 3.5 mm coronary angioplasty balloon was delivered and deployed (Fig. 2b), and then exchanged for a similar 5 mm angioplasty balloon in order to serially dilate the fenestration in the membrane. Two 6 mmx22 mm SIGBI G SETA® covered stents, (Latecba. SA. Buenos Aires. Argentina) were placed through the stent graft membrane fenestration and deployed in the left renal artery, thus, restoring true lumen flow and closing the re-entry tear at this level (Fig. 2 c).

Finally, two balloon expandable limb extensions RIK SETA® (Latecba.SA. Buenos Aires. Argentina) were deployed in each of the common iliac arteries, using “kissing stent” technique. An additional extension, also balloon-expandable RIK F SETA® (Latecba.SA. Buenos Aires. Argentina), was implanted in the left external iliac artery. This RIK F module had a fenestration at the origin of the hypogastric artery, to allow the permeability of this vessel. The total time was 330 minutes, 420 cc of non-ionic contrast were used and the mean fluoroscopy time was 93 minutes. The patient tolerated the procedure well without any paraplegia or other adverse event. Spinal drain was removed on post-operative day 1. He was discharged on day 4.

**Fig. 3:**
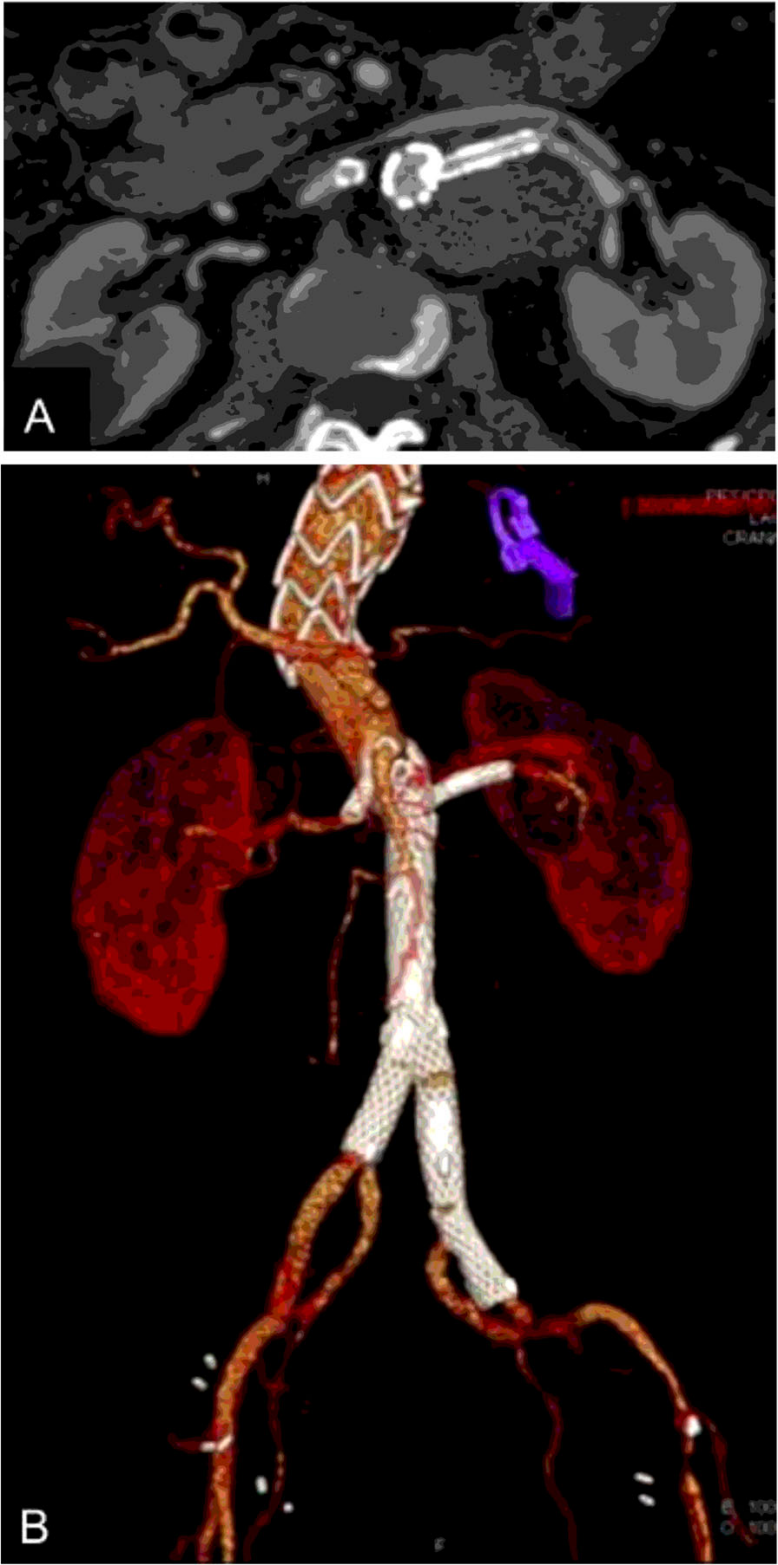
**a** Axial contrast-enhanced image showing patency of the left renal connection stent with complete thrombosis of the aortic aneurysm. **b** 3D reconstruction showing patency of all splanchnic arteries, reconstitution of flow through de TL and patency of both hypogastric arteries

A contrast-enhanced CTA image performed at 30 days showed patency of both renal arteries and superior mesenteric artery, absence of endoleaks, and complete occlusion of the false lumen and restoration of true lumen flow in all visceral vessels (Fig. 3).

## Discussion and conclusion

20–40 % of patients suffering from acute thoraco-abdominal type B aortic dissection present with aneurysmal dilatation of the false lumen during follow up [1, 3]. Both conventional open and hybrid open-endovascular surgery are valid therapeutic alternatives but can be associated with high morbidity and mortality even in experienced surgical centers [2, 4].

Complete endovascular treatment of chronic abdominal aortic dissection with aneurysmal degeneration using fenestrated or branched stents has a number of limitations. The small diameter of the TL and often immobile septum make endovascular repair challenging with conventional stent grafts. Moreover, sealing the re-entry tears in the FL that often involve the celiac trunk and the lower renal artery is often not possible [5].

The use of endovascular occluders to try to seal the re-entry into the FL has been associated with poor outcomes by some; notably the persistence of endoleaks and the absence of remodelling of the aortic lumen [6, 7].

The SETA MUG® is a non-tapered endoprosthesis, full stented, with no bare metal proximal stents (‘free flow’) and consisting of a multilayer membrane and hybrid stent design with 2 types of cells. It is mounted on a 25 mm diameter balloon, for this case. The high porosity of the multilayer membrane allows for in situ fenestration thus allowed us to seal the FL re-entry tear at the level of the left renal artery and restore true lumen flow to the kidney. Importantly, this membrane is easily visible and permits one orient the crossing wire/catheter precisely at the level of the renal ostia and thus puncture the membrane and accurately deploy the bridging stent-graft without using 3D image fusion. Regarding the long-term evolution of FL, in a recent meta-analysis, P Qui et al. reported the results of 8 observational studies in which 914 patients participated, where the PETTICOAT technique does not favor aortic remodeling due to the absence of false lumen thrombosis [8].

We believe that the SETA MUG® device would also be favorable for the remodeling of the abdominal aorta, similar to that reported with the E-PETTICOAT technique [9].

**Fig. 4:**
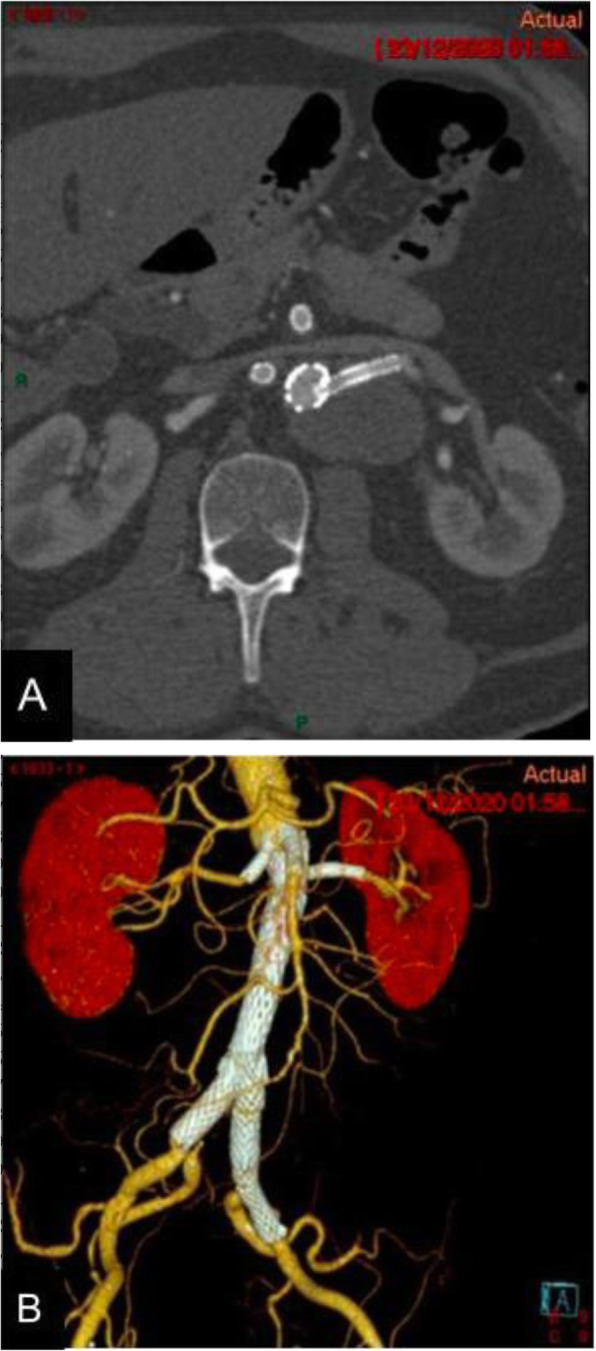
Eight months follow-up. **a** Axial contrast-enhanced image showing the left renal branch without re- stenosis and decrease in size of the residual aneurysmal sac. **b** 3D reconstruction showing patency of all abdominal aortic branches as well as both hypogastric arteries

During the 8-month follow-up, the patient remained asymptomatic and the CTA showed patency of all branches as well as the left hypogastric artery with complete occlusion and reduction in the size of the FL (Fig. 4).

We did not find previous reports in the international literature regarding a stent with such characteristics. As an antecedent to this endoprosthesis, we carried out a research work in 7 sheep at the Veterinary University of Zaragoza, the results of which are in the process of being published.

To guarantee blood flow to the superior mesenteric artery and right renal artery covered stents were used (chimney technique), taking into consideration that we expected no endoleak due to the fact that the lumen of the suprarenal aorta was non-aneurysmal [10] and also, due to the initial learning curve with the SETA MUG® device.

Creatinine levels immediately after the intervention and the previous control prior to the performance of the computed tomography revealed no changes.

While cardiac and renal morbidity is relatively low, the risk of spinal cord ischemia remains significantly high during endovascular repair of thoraco-abdominal aneurysms, and therefore highlights the importance of staging these procedures when possible [11].

In conclusion, this first case demonstrates the feasibility of the SETA MUG device design in which in-situ fenestration of the stent graft membrane can be performed to treat aortic dissection with persistent false lumen flow. Pending further studies, this may soon become a new option in the endovascular therapy for patients with chronic abdominal aortic dissection such as our patient, as well as those patient anatomies with difficult landing zones (short and/or conical necks, etc.) or those with type1 A endoleaks, who might otherwise require fenestrated stent grafts or fenestrated cuffs. Larger studies with longer follow-up are essential to fully evaluate the safety and effectiveness of this technology and the need of possible secondary procedures.

Approved by Leben Clinic ethics committee.

## Data Availability

all data related to the MUG device are property of the company Latecba. SA.
